# Oral intake of phenylbutyrate with or without vitamin D_3_ upregulates the cathelicidin LL-37 in human macrophages: a dose finding study for treatment of tuberculosis

**DOI:** 10.1186/1471-2466-13-23

**Published:** 2013-04-16

**Authors:** Akhirunnesa Mily, Rokeya Sultana Rekha, S M Mostafa Kamal, Evana Akhtar, Protim Sarker, Zeaur Rahim, Gudmundur H Gudmundsson, Birgitta Agerberth, Rubhana Raqib

**Affiliations:** 1 International Centre for Diarrheal Disease Research, Bangladesh (icddr,b), Mohakhali, Dhaka, 1212, Bangladesh; 2 Department of Medical Biochemistry and Biophysics, Karolinska Institutet, Stockholm, 17177, Sweden; 3 National Institute of the Diseases of the Chest and Hospital, Mohakhali, Dhaka, 1212, Bangladesh; 4 Institute of Biology, University of Iceland, Reykjavik, 101, Iceland

**Keywords:** Innate immunity, Antimicrobial peptides, Monocyte derived macrophages, *Mycobacterium*

## Abstract

**Background:**

We earlier showed that 4-phenylbutyrate (PB) can induce cathelicidin LL-37 expression synergistically with 1,25-dihydroxyvitamin D_3_ in a lung epithelial cell line. We aimed to evaluate a therapeutic dose of PB alone or in combination with vitamin D_3_ for induction of LL-37 expression in immune cells and enhancement of antimycobacterial activity in monocyte-derived macrophages (MDM).

**Methods:**

Healthy volunteers were enrolled in an 8-days open trial with three doses of PB [250 mg (Group-I), 500 mg (Group-II) or 1000 mg (Group-III)] twice daily (b.d.) together with vitamin D_3_ {5000 IU once daily (o.d.)}, PB (500 mg b.d.) (Group-IV) or vitamin D_3_ (5000 IU o.d.) (Group-V), given orally for 4 days. Blood was collected on day-0, day-4 and day-8; plasma was separated, peripheral blood mononuclear cells (PBMC), non-adherent lymphocytes (NAL) and MDM were cultured. LL-37 transcript in cells and peptide concentrations in supernatant were determined by qPCR and ELISA, respectively. In plasma, 25-hydorxyvitamin D_3_ levels were determined by ELISA. MDM-mediated killing of *Mycobacterium tuberculosis (Mtb)* (H37Rv) was performed by conventional culture method.

**Results:**

MDM from Group-II had increased concentration of LL-37 peptide and transcript at day-4, while Group-I showed increased transcript at day-4 and day-8 compared to day-0 (p < 0.05). Both Group-I and -II exhibited higher levels of transcript on day-4 compared to Group-III and Group-V (p < 0.035). Increased induction of peptide was observed in lymphocytes from Group-II on day-4 compared to Group-I and Group-IV (p < 0.05), while Group-IV showed increased levels on day-8 compared to Group-I and Group-III (p < 0.04). Intracellular killing of *Mtb* on day-4 was significantly increased compared to day-0 in Group-I, -II and -V (p < 0.05).

**Conclusion:**

The results demonstrate that 500 mg b.d. PB with 5000 IU o.d. vitamin D_3_ is the optimal dose for the induction of LL-37 in macrophages and lymphocytes and intracellular killing of *Mtb* by macrophages. Hence, this dose has potential application in the treatment of TB and is now being used in a clinical trial of adults with active pulmonary TB (NCT01580007).

## Background

Tuberculosis (TB), caused by *Mycobacterium tuberculosis* (*Mtb*), is a predominant public health problem worldwide and responsible for about 3 million deaths annually [1]. The prevalence of TB is increasing due to the spread of antibiotic-resistant strains of *Mtb*[2,3]. The deleterious consequences of co-infection with HIV is the emergence of XDR (extremely drug resistance) cases in Africa and other countries in recent years [4]. Advances in anti-tuberculous therapies and alternative treatment strategies are urgently required for the treatment of TB patients and exposed individuals at high risk of developing TB [5,6].

Antimicrobial peptides (AMPs) are important effectors of the innate defense system [7]. AMPs can limit growth and virulence properties of microbes directly or indirectly by enhancing the host immune system. Induction of endogenous AMP expression by administration of extrinsic compounds may be an attractive approach for alternative therapy in combating infectious diseases. Cathelicidins [8] and Defensins [9] and are major classes of AMPs, in mammals LL-37 is the only human cathelicidin and is expressed by both circulating white blood cells and epithelial surfaces including lungs [10].

The active form of vitamin D_3_ was shown to regulate the production of AMPs, i.e. cathelicidin LL-37, which plays an important role in the innate immune defense against infections including TB [11,12]. These studies have encouraged many investigators to initiate vitamin D trials in TB patients with renewed enthusiasm [5,13-15] (http://NCT01130311; http://NCT00507000; http://NCT00366470; http://NCT00677339; http://NCT00918086). In a systemic review and meta-analysis of observational studies, evidence of an association with vitamin D_3_ deficiency and active TB was demonstrated [16].

Our group has earlier shown that cathelicidin is downregulated in the mucosal epithelia during acute diarrhea [17,18] and in infection with *Neisseria gonorrhea*[19]. We showed that the downregulation of the rabbit cathelicidin (CAP-18) in the large intestine can be counteracted by oral treatment with sodium butyrate, a short chain fatty acid and phenylbutyrate (PB), an analogue of butyrate in experimental model of shigellosis [17,20]. PB further counteracted the downregulation of CAP-18 in the lung and tracheal epithelia through systemic effects [20]. We further demonstrated that PB can induce LL-37 expression synergistically with 1,25-dihydroxyvitamin D_3_ at both protein and mRNA levels in a lung epithelial cell line [21]. It is thus likely that oral supplementation with PB will boost innate immunity in the lung mucosa by increasing expression of innate defense proteins. A dose of PB corresponding to that used for rabbits [20] for induction of cathelicidin was calculated by allometric scaling for use in adults. The calculations suggested that an effective dose for a 60 kg human would be approximately 600 mg of PB twice daily.

Our aim in this study was to determine the optimal dose of PB with or without vitamin D_3_ in adults for induction of LL-37 in immune cells and increase in functional capacity of macrophages in killing of *Mtb*. Thus, healthy adult volunteers were treated with different doses of PB and in combination with a defined concentration of vitamin D_3_.

## Methods

### Study design

Healthy participants (age range 18 to 55 yrs; n = 15) working at International Centre for Diarrheal Disease Research, Bangladesh (icddr,b) and National Institute of the Diseases of the Chest and Hospital (NIDCH), Dhaka, Bangladesh were recruited in this study. The study was approved by the Ethical Review Committee of icddr,b. Informed consent was obtained from the participants after explaining the nature and purpose of the study. Individuals were excluded if they had symptoms or clinical signs of infection, e.g. fever or diarrhea within the last 2–3 months, if taking corticosteroids, diuretics, or supplementary vitamin D_3_ (either alone or as part of a multivitamin preparation). The volunteers were asked to abstain from taking any vitamins or supplements for at least two weeks prior to and during the study period. There were five groups, each consisting of 3 participants. Groups-I, -II and -III received the following doses respectively: 250 mg, 500 mg or 1 g of PB b.d. plus 5000 IU vitamin D_3_ o.d. for 4 consecutive days. Group-IV received 500 mg PB b.d. and Group-V received 5000 IU vitamin D_3_ o.d. for 4 consecutive days. All healthy volunteers were followed for another 4 days after the treatment to monitor possible side effects. Four-phenylbutyrate (Tributyrate) and the placebo tablet were obtained from Fyrklövern Scandinavia AB, Mönsterås, Sweden; vitamin D_3_ (Vigantol oil) and the placebo oil were obtained from Merck KGaA, Darmstadt, Germany. The healthy volunteer trial was conducted to determine an optimal dose for the clinical trial in TB patients (http://NCT01580007).

### Blood collection and cell culture

Peripheral blood was collected at 3 time points *(*day-0, day-4 and day-8) from each participant (25 mL/individual). Peripheral blood mononuclear cells (PBMCs) were isolated from whole blood by Ficoll-Hypaque density gradient centrifugation and plasma was collected and stored at −20°C for later measurement of 25-hydroxyvitamin D_3_, calcium, (serum glutamate pyruvate transaminase (SGPT; liver function marker) and creatinine (kidney function marker). The PBMC pellet was washed and suspended in a culture medium containing 10% autologous plasma in RPMI-1640, 1% L-glutamine, 1% sodium pyruvate, 0.5% amphotericin B and 1% penicillin-streptomycin (Gibco, Grand Island, NY, USA) and plated in two parallel 4-well tissue culture plates (NUNC, Roskilde, Denmark). One tissue culture plate was utilized for analyses of LL-37 peptide and transcript and the other plate was used for macrophage-mediated *Mtb* killing assay.

PBMCs were counted and incubated in culture plates for 3 days. Thereafter, supernatant containing the non-adherent cells (mostly lymphocytes, 80-90%) was removed, centrifuged to collect the clear supernatant which was the extracellular fluid (ECF) of PBMC. The non-adherent lymphocytes were treated with saponin for 10 minutes, centrifuged and supernatant collected as intracellular fluid (ICF) from lymphocytes. Supernatant and cell pellet were stored for further analysis. The remaining adherent cells in the culture plate were MDM, which were harvested and treated with saponin. After centrifugation, supernatant (ICF) and cells were stored until further analysis. RNALater (Qiagen GmbH, Hilden, Germany) was added to the cell pellet for RNA extraction. In another experiment, one part of PBMC was stimulated ex vivo with Bacille Calmette-Guerin, (BCG, 10 μg/mL: Japan BCG Laboratory, Tokyo, Japan) for 3 days, supernatant containing the non-adherent cells was removed, centrifuged to collect the clear supernatant which was the ECF of BCG stimulated PBMC.

### Vitamin D_3_, calcium, albumin, SGPT and creatinine in plasma

In plasma, 25-hydroxyvitamin D_3_ was measured by a commercial ELISA kit (IDS, Fountain Hills, Arizona, USA) that determines 25-hydroxyvitamin D_3_ (100%), 25-hydroxyvitamin D_2_ (75%) and 24,25-dihydroxyvitamin D_3_ (≥100%). Calcium, albumin and creatinine were assessed by Roche automated clinical chemistry analyzer, Hitachi 902. Serum glutamate-pyruvate transaminase (SGPT) was measured by Beckman-Coulter AU680 (Japan).

### LL-37 ELISA

LL-37 levels in ICF of macrophage and lymphocyte lysates as well as in ECF of PBMC and BCG stimulated PBMC were measured by ELISA. Duplicate samples were tested and concentrations were calculated using a standard curve generated from synthetic LL-37 (Innovagen, Lund, Sweden). Brief procedure of ELISA is as follow. Polystyrene microtiter plates (Maxisorp by NUNC, Naperville, IL, USA) were coated with monoclonal anti-LL-37 [22] (5 μg/ml) in carbonate buffer (15 mM sodium carbonate, 35 mM sodium bicarbonate and 0.02% sodium azide [pH 9.6]) and incubated overnight at 4°C. After washing, non-specific binding was blocked with 0.1% gelatin in tris-buffered saline (pH 7.5) for 1 hour at RT. Standards and samples were then added and incubated overnight at 4°C. Biotinylated rabbit anti-LL-37 (1 μg/mL) (Innovagen) was added and incubated for 2 h at room temperature (RT), followed by the incubation with Streptavidin-alkaline phosphatase conjugate (Chemicon, Melbourne, Australia) for another 2 h at RT. Four-methylumbelliferyl phosphate was used as substrate (Molecular Probes, Europe BV, Leiden, The Netherlands) and fluorescence was measured at an excitation wavelength of 360 nm and emission wavelength of 450 nm.

### Quantitative real time RT-PCR amplification of LL-37 mRNA

RNA was extracted from macrophages and lymphocytes utilizing RNeasy Mini kit as described by the manufacturer (Qiagen GmbH). cDNA was synthesized using Superscript III First-Strand Synthesis System (Invitrogen, Grand Island, NY, USA). The CAMP gene encoding transcript LL-37 relative to the housekeeping 18S rRNA was measured in triplicate from the cDNA samples by real-time quantitative RT-PCR using CFX96 Real-Time PCR Detection Systems (Bio-Rad,) and the 18 s rRNA–housekeeping gene kit (Applied Biosystems, Foster City, CA, USA). The sequences of forward and reverse primers for LL-37 transcript were 5’-TCACCAGAGGATTGTGACTTCAA-3’ and 5’-TGAGGGTCACTGTCCCCATAC-3’, respectively (Primer Express; Applied Biosystems). The results were analyzed by using the relative standard method [19].

### Macrophage mediated killing of *Mycobacterium tuberculosis*

MDM in the culture plates were infected with previously prepared log phased *Mtb* H37Rv strain (from Tuberculosis Research Center, Chennai, India) in culture medium without antibiotics [23]. Initially 3 different multiplicity of infection (MOI; 1:10; 1:25 and 1:50) were tested and the ratio of 1:25 was found to be the optimal MOI, resulting in a clear bacterial killing. After 2 hours of exposure the culture plates were washed 3 times with warm RPMI to remove the extracellular bacteria and the infected MDM were cultured for three additional days in a medium with autologous plasma and antibiotics (penicillin-streptomycin, amphotericin B) (Gibco). Thereafter, the cells were lysed with 0.3% saponin-PBS and vigorous pipetting to collect viable intracellular *Mtb*. The lysates were cultured on Middle Brook 7H11 agar medium supplemented with 10% Middle Brook OADC (oleic acid, albumin, dextrose and catalase) enrichment (Becton Dickinson, Sparks, MD, USA). Bacterial viability was calculated by counting colony forming units (CFU) on agar plates after 21–28 days of culture at 37°C. Before initiation of any intervention, the day-0 MDM-mediated killing served as the control for all groups.

### Statistical analysis

Statistical analyses were performed using SigmaStat 3.1 for Windows (Systat Software Inc., Point Richmond, CA, USA) and SPSS 17.0 for Windows (SPSS Inc, Chicago, Illinois, USA). Results were expressed as mean with standard deviation. Sex and age was matched for each group. Data distribution patterns were checked by using scatter plots, and normality and homogeneity of variances were checked by descriptive statistics. Two-way repeated measure ANOVA was performed when both the sphericity and normality of the data was met. When condition for sphericity was violated or normality of data failed, one Way Analysis of Variance (ANOVA) was performed followed by the Tukey multiple comparison test for between groups and within group variables. Kruskal-Wallis ANOVA on Ranks was performed when data was not normally distributed. Analysis of Co-variance was performed when data at entry level was significantly different. P-values < 0.05 were considered significant.

## Results

### Plasma levels of vitamin D_3_ and calcium

**Figure 1:**
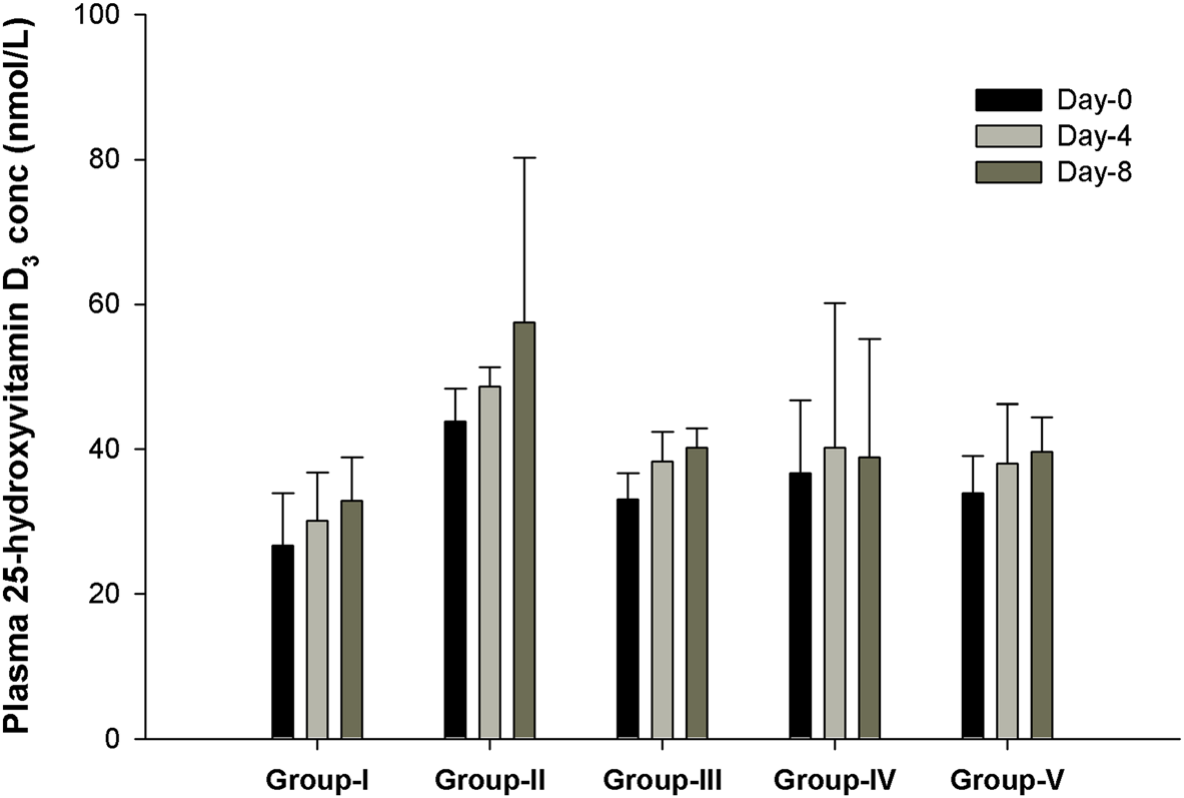
**Plasma 25-hydroxyvitamin D**_**3 **_**concentration in healthy adults before and after supplementation.** Group-I: 250 mg PB b.d. + 5000 IU vitamin D_3_ o.d.; Group-II: 500 mg PB b.d. + 5000 IU vitamin D_3_ o.d.; Group-III: 1000 mg PB b.d. + 5000 IU vitamin D_3_ o.d.; Group-IV: 500 mg PB b.d.; Group-V: 5000 IU vitamin D_3_ o.d. PB: Phenylbutyrate. b.d.: twice daily. o.d. : once daily. Data were analyzed by One-way ANOVA.

The average of 25-hydroxyvitamin D_3_ levels in plasma increased in all groups except for Group-IV (treated with only PB). However, the increase was not significant (Figure 1). Sex and age was matched for each group. Similarly, no changes were observed in albumin-adjusted calcium levels during the study period in any of the groups (data not shown).

SGPT level before treatment was normal in all healthy volunteers except for one male volunteer in Group-I, who had 66.49 IU/L that was above the normal upper range of 56 IU/L. However, the SGPT level (12.08 IU/L) declined on day-4 to normal level. None of the volunteers showed any increase in SGPT level after ingestion of PB alone or in combination with vitamin D_3_ on day-4 or −8 (Additional file 1: Table S1).

### Synergistic effects of PB and vitamin D_3_ on LL-37 expression in macrophages

Significant differences in concentration of MDM derived LL-37 transcripts and peptides were observed within days. A significant increase in both peptide (p = 0.05) and transcript (p = 0.011) was noted in Group-II in day-4 compared to day-0 (Table 1 and 2). Group-I showed significantly higher levels of LL-37 transcript but not peptide at day-4 (p = 0.015) and day-8 (p = 0.042) compared to day-0. Group-IV showed a marginal increase in LL-37 transcript levels on day-4 compared to day-0 (p = 0.07) (Table 1).

**Table 1:** Expression of LL-37 transcript in monocyte-derived macrophages from healthy adults before and after supplementation

		**LL-37 transcript (copy no.)**		
		**Day-0**	**Day-4**	**Day-8**
Group-I	MDM	9.68 ± 9.0	64.37 ± 19.03^a 1,2^	21.61 ± 18.71^b^
Group-II	MDM	16.24 ± 1.77	62.42 ± 19.56^c 3,4^	38.25 ± 15.65
Group-III	MDM	10.94 ± 2.01	10.40 ± 16.19	41.05 ± 52.30
Group-IV	MDM	8.71 ± 4.64	33.87 ± 16.76	13.42 ± 9.57
Group-V	MDM	10.13 ± 5.41	11.54 ± 12.90	8.74 ± 6.27

Note: Group-I: 250 mg PB b.d. + 5000 IU vitamin D_3_ o.d.; Group-II: 500 mg PB b.d. + 5000 IU vitamin D_3_ o.d.; Group-III: 1000 mg PB b.d. + 5000 IU vitamin D_3_ o.d.; Group-IV: 500 mg PB b.d.; Group-V: 5000 IU vitamin D_3_ o.d.. Data expressed as Mean ± Standard Deviation. One Way Analysis of Variance (ANOVA) method was used for statistical analysis. Differences are significant, when p < 0.05. Difference between, ^a^day-4 and day-0 within Group-I (p = 0.01); ^b^day-8 and day-0 within Group-I (p = 0.04); ^c^day-4 and day-0 within Group-II (p = 0.01); ^1^Group-I and -III within day-4 (p = 0.02); ^2^Group-I and -V within day-4 (p = 0.034); ^3^Group-II and -III within day-4 (p = 0.031); ^4^Group-II and -V within day-4 (p = 0.035). *PB*, Phenylbutyrate; *MDM*, Monocyte-derived macrophages; *b.d*., Twice daily; *o.d*., Once daily.

**Table 2:** Expression of LL-37 peptide in monocyte-derived macrophages from healthy adults before and after supplementation

		**LL-37 level in ICF (ng/million cells)**		
		**Day-0**	**Day-4**	**Day-8**
Group-I	MDM	0.52 ± 0.06	0.72 ± 0.28	0.68 ± 0.19
Group-II	MDM	0.33 ± 0.02	1.22 ± 0.49^a 1^	0.36 ± 0.05
Group-III	MDM	0.28 ± 0.00	0.27 ± 0.02	0.27 ± 0.02
Group-IV	MDM	0.15 ± 0.02	0.13 ± 0.02	0.16 ± 0.02
Group-V	MDM	0.21 ± 0.08	0.20 ± 0.05	0.23 ± 0.09

Note: Group-I: 250 mg PB b.d. + 5000 IU vitamin D_3_ o.d.; Group-II: 500 mg PB b.d. + 5000 IU vitamin D_3_ o.d.; Group-III: 1000 mg PB b.d. + 5000 IU vitamin D_3_ o.d.; Group-IV: 500 mg PB b.d.; Group-V: 5000 IU vitamin D_3_ o.d. Data expressed as Mean ± Standard Deviation. One Way Analysis of Variance (ANOVA) method was used for statistical analysis. Kruskal-Wallis ANOVA on Ranks was performed when the data was not normally distributed; analysis of Co-variance was performed when significant difference was found at entry level. Differences are significant, when p < 0.05. Difference between, ^a^day-4 and day-0 within Group-II (p = 0.02); ^1^Group-II and -III within day-4 (p = 0.034). *ICF*, Intracellular fluid; *PB*, Phenylbutyrate; *MDM*, Monocyte-derived macrophages; *b.d*., Twice daily; *o.d*., Once daily.

When comparing between groups, Group-I and Group-II exhibited significantly higher levels of LL-37 transcript on day-4 compared to Group-III (p = 0.025 and p = 0.03 respectively) and Group-V (p = 0.03 and p = 0.035 respectively) (Table 1). Furthermore, Group-II showed higher levels of peptide compared to Group-IV (p = 0.01) (Table 2).

### PB and vitamin D_3_ induce LL-37 expression in lymphocytes

Lymphocyte-derived LL-37 transcript did not show any significant induction within days (Table 3). However, LL-37 peptide induction on day-4 was significantly higher in Group-II compared to Group-I (p = 0.03) and Group-IV (p = 0.05) (Table 4). Group-IV showed increased production of peptide on day-8 compared to Group-III (p = 0.03) and Group-I (p = 0.04).

**Table 3:** Expression of LL-37 transcript in non-adherent lymphocytes in healthy adults before and after supplementation

		**LL-37 transcript (copy no.)**		
		**Day-0**	**Day-4**	**Day-8**
Group-I	NAL	9.60 ± 2.80	19.5 ± 7.65	7.46 ± 5.00
Group-II	NAL	29.57 ± 6.17	54.43 ± 16.48	38.25 ± 20.66
Group-III	NAL	19.33 ± 8.21	30.00 ± 10.60	20.00 ± 5.01
Group-IV	NAL	14.78 ± 3.08	18.68 ± 5.92	19.12 ± 10.33
Group-V	NAL	9.18 ± 2.39	23.30 ± 14.58	20.71 ± 7.45

Note. Group-I: 250 mg PB b.d. + 5000 IU vitamin D_3_ o.d.; Group-II: 500 mg PB b.d. + 5000 IU vitamin D_3_ o.d.; Group-III: 1000 mg PB b.d. + 5000 IU vitamin D_3_ o.d.; Group-IV: 500 mg PB b.d.; Group-V: 5000 IU vitamin D_3_ o.d. Data expressed as Mean ± Standard Deviation. One Way Analysis of Variance (ANOVA) method was used. Analysis of Co-variance was performed when significant difference was found at entry level. *PB*, Phenylbutyrate; *NAL*, Non-adherent lymphocytes; *b.d*., Twice daily; *o.d*., Once daily.

**Table 4:** Expression of LL-37 peptide in non-adherent lymphocytes in healthy adults before and after supplementation

		**LL-37 level in ICF (ng/million cells)**		
		**Day-0**	**Day-4**	**Day-8**
Group-I	NAL	0.24 ± 0.07	0.23 ±0.11	0.18 ± 0.12
Group-II	NAL	0.28 ± 0.04	0.49 ± 0.16 ^1,2^	0.33 ± 0.11
Group-III	NAL	0.31 ± 0.11	0.25 ± 0.02	0.17 ± 0.06
Group-IV	NAL	0.27 ± 0.14	0.21 ± 0.02	0.68 ± 0.32 ^3,4^
Group-V	NAL	0.19 ± 0.08	0.37 ± 0.07	0.27 ± 0.15

Note: Group-I: 250 mg PB b.d. + 5000 IU vitamin D_3_ o.d.; Group-II: 500 mg PB b.d. + 5000 IU vitamin D_3_ o.d.; Group-III: 1000 mg PB b.d. + 5000 IU vitamin D_3_ o.d.; Group-IV: 500 mg PB b.d.; Group-V: 5000 IU vitamin D_3_ o.d. Data expressed as Mean ± Standard Deviation. One Way Analysis of Variance (ANOVA) method was used for statistical analysis. Differences are significant, when p < 0.05. Difference between, ^1^Group-II and -I within day-4 (p = 0.05); ^2^Group-II and -IV within day-4 (p = 0.03); ^3^Group-IV and -I within day-8 (p = 0.04); ^4^Group-IV and-III within day-8 (p = 0.036). *ICF,* Intracellular fluid; *PB*, Phenylbutyrate; *NAL*, Non-adherent lymphocytes; *b.d.*, Twice daily; *o.d*., Once daily.

### No effects of PB and vitamin D_3_ on LL-37 expression in PBMC

There was no significant induction of LL-37 production in ECF of total PBMC within days or between groups. Stimulation of PBMC with live BCG also did not show any remarkable increase in LL-37 release in the ECF (0.53 ± 0.17 ng/10^6^ PBMC) compared to the ECF of unstimulated PBMC (0.50 ± 0.17 ng/10^6^ PBMC) (p = 0.92).

### PB and/or vitamin D_3_ mediate enhanced killing of *Mtb ex vivo*

**Figure 2:**
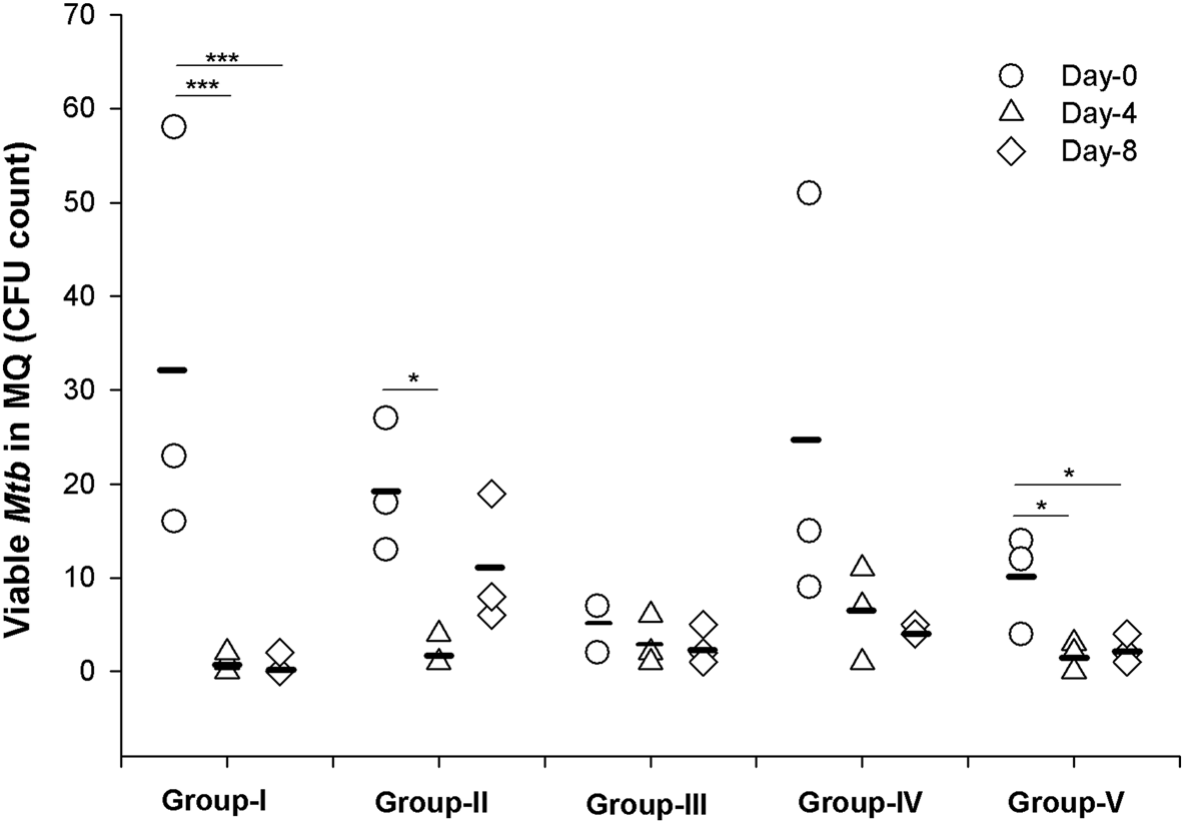
**Viable *Mtb *CFU count in Monocyte derived macrophages (MDM).** MDM from different group of volunteers were incubated with *Mtb* H37Rv strain for 2 h, after that extracellular bacteria were removed and cultured for 3 days, cells were lysed and plated for variable colony (CFU) count. Group-I: 250 mg PB b.d. + 5000 IU vitamin D_3_ o.d.; Group-II: 500 mg PB b.d. + 5000 IU vitamin D_3_ o.d.; Group-III: 1000 mg PB b.d. + 5000 IU vitamin D_3_ o.d.; Group-IV: 500 mg PB b.d.; Group-V: 5000 IU vitamin D_3_ o.d.. PB: Phenylbutyrate. b.d.: twice daily. o.d. : once daily. The straight horizontal line indicates means. Data were analyzed by two-way (treatment and time) repeated measures ANOVA. * p < 0.05, and *** p < 0.001.

Group-II demonstrated significantly higher intracellular killing of *Mtb* by MDM at day-4 (p = 0.027) compared to day-0. Group-I and -V exhibited a significant increase in intracellular killing on day-4 (p < 0.001 and p = 0.019 respectively) and day-8 (p < 0.001 and p = 0.051 respectively) compared to day-0 (Figure 2). The other Groups did not show any increase in the killing activity of MDM (Figure 2).

## Discussion

In this study we demonstrated that oral supplementation of healthy adult volunteers with 500 mg PB b.d. plus 5000 IU vitamin D_3_ o.d. (Group-II) consistently induced LL-37 in both macrophages and lymphocytes, and also exhibited increased MDM derived intracellular killing of *M. tuberculosis.*

In an animal model of shigellosis we have previously shown that *Shigella* infection causes downregulation of the rabbit cathelicidin CAP-18 in the epithelia of rectum, lung and trachea [17,20]. Oral feeding of butyrate (0.14 mmol/dose) or PB (0.14 mmol/dose) up-regulates CAP-18 expression in the lung and rectal epithelia of these rabbits and reduces shedding of *Shigella* in stool [20]. We have also demonstrated that PB, induces the *CAMP* gene expression synergistically with 1,25-dihydroxyvitamin D_3_ at both protein and mRNA levels in the VA10 lung epithelial cell line [21]. We have now further shown in humans, that oral intake of PB alone or in combination with vitamin D_3_ can also induce LL-37 in immune cells. In this study, three potential doses of PB were selected for healthy adults. Group I (250 mg b.d. with 5000 IU vitamin D_3_ o.d.) showed significant increase in LL-37 transcript but not peptide in MDM; no changes in peptide or transcript was noted in NAL. Group-II (500 mg b.d. plus 5000 IU vitamin D_3_ o.d.) showed increase in both peptide and transcript in MDM and peptide in NAL. In Group-III with the higher dose of PB (1 g b.d.) along with vitamin D_3_ there was no increase in LL-37 transcript or peptide in cells after PB supplementation. Butyrate is known to inhibit RNA and protein synthesis at high concentrations [24-26]. We may speculate that in a similar fashion PB at high doses is inhibitory to the expression of LL-37 both at transcriptional and translational levels. The dose of Group-II appears superior in inducing both peptide and mRNA concentrations in MDM and peptide in lymphocytes.

Various cell types including T lymphocytes are known to express LL-37 [27,28]. However, to our knowledge this is the first study to report enhanced *in vivo* induction of LL-37 in lymphocytes after oral supplementation with PB alone or in combination with vitamin D_3_. This finding thus underscores the significance of PB therapy in TB infection since T cells play a major role in the host defense against tuberculosis [29].

The present study showed that oral intake of vitamin D_3_ (Group-V) alone or in combination with PB (Group-I and -II) exhibited a marked increase in intracellular killing of *Mtb*. Vitamin D_3_-induced LL-37 expression is an important factor in fighting TB [11,12]. Killing of *Mtb* by macrophages is directly correlated with *CAMP* gene expression encoding LL-37 and plasma levels of 25-hydroxyvitamin D_3_[12]. Furthermore, it was shown that activation of Toll-Like Receptor 2/1 in human macrophages up-regulated expression of the genes encoding vitamin D receptor and vitamin D-hydroxylase, leading to induction of LL-37 with subsequent killing of intracellular *Mtb*[11]. Recent studies have shown that human cathelicidin is a key mediator of 1,25-dihydroxyvitamin D_3_-induced autophagy and hence provide a mechanistic insight into the role of cathelicidin in combating *Mtb*[30,31]. Interestingly, *in vitro* studies have shown that short chain fatty acids, butyrate and propionate can induce both apoptosis and autophagy [32]. PB is an analogue of butyrate and it is quite likely that PB in combination with Vitamin D_3_ may also induce autophagy and LL-37 mediated killing of *Mtb ex vivo*.

The major limitation of this study is the small sample size and the short duration of the PB and vitamin D_3_ supplementation. The lack of increase in plasma 25-hydroxyvitamin D_3_ level after 4 days supplementation was not unexpected since 1–2 months supplementation is needed to reach steady state vitamin D_3_ levels.

## Conclusion

In conclusion, oral combination dose of 500 mg PB b.d. with 5000 IU vitamin D_3_ o.d. appears to be the optimum dose to induce both LL-37 peptide and transcript expression in functional immune cells as well as enhance intracellular *Mtb* killing in macrophages. The dose of 250 mg PB b.d. with 5000 IU vitamin D_3_ o.d. also increased intracellular killing however it enhanced only LL-37 transcript levels (not peptide) in macrophages but not in lymphocytes. This pilot study has generated results for a potential dose in a clinical trial of adults with active pulmonary TB (http://NCT01580007).

### Ethical approval

The study was approved by the Ethical Review Committee of International Centre for Diarrheal Disease Research, Bangladesh (icddr,b).

## Abbreviations

AMPs: Antimicrobial peptides; b.d.: Twice daily; CFU: Colony forming units; ECF: Extracellular fluid; HDAC: Histone deacetylase; ICF: Intracellular fluid; MDM: Monocyte-derived macrophages; Mtb: *Mycobacterium tuberculosis*; o.d.: Once daily; PB: 4-Phenylbutyrate; PBMCs: Peripheral blood mononuclear cells; TB: Tuberculosis.

## Competing interests

Birgitta Agerberth, Gudmundur H. Gudmundsson and Rubhana Raqib have a pending patent application for the use of phenylbutyrate to the treatment of infections. There is no financial arrangement between any of the authors.

## Authors’ contributions

RR, BA contributed to the conception and design of the study; AM, RSR, EA and PS performed laboratory analysis, AM, RSR and RR performed the statistical analysis; AM, RSR and RR prepare the first draft of the manuscript. BA, GHG, ZR, SMMK and PS revised the manuscript. All authors read and approved the final version of the manuscript.

## Pre-publication history

The pre-publication history for this paper can be accessed here:

http://www.biomedcentral.com/1471-2466/13/23/prepub

## Supplementary Material

Additional file 1: Table 1 Serum glutamate-pyruvate transaminase and creatinine levels in healthy adults supplemented with phenylbutyrate and vitamin D3 alone or in combination in different doses. Click here for file
