# Supplementary material for: Oral intake of phenylbutyrate with or without vitamin D3 upregulates the cathelicidin LL-37 in human macrophages: a dose finding study for treatment of tuberculosis
Source: BMC Pulm Med. 2013 Apr 16;13:23. doi: 10.1186/1471-2466-13-23 (PMC3637063; doi:10.1186/1471-2466-13-23)
Supplement: Additional file 1: Table 1 — Serum glutamate-pyruvate transaminase and creatinine levels in healthy adults supplemented with phenylbutyrate and vitamin D3 alone or in combination in different doses. [file 1471-2466-13-23-S1.pdf]

Table S1. Serum glutamate-pyruvate transaminase and creatinine levels in healthy adults supplemented with phenylbutyrate and vitamin D_3 alone or in combination in different doses**.**

	Markers	Day-0	Day-4	Day-8
Group-I	SGPT	26.6 ± 17.8	35.5 ± 20.8	32.0 ± 16.0
	Creatinine	57.7 ± 5.8	60.6 ± 7.4	63.6 ± 6.1
Group-II	SGPT	31.0 ± 31.2	24.2 ± 26.1	23.3 ± 26.0
	Creatinine	64.2 ± 8.7	64.7 ± 7.8	60.3 ± 7.7
Group-III	SGPT	15.3 ± 4.5	17.9 ± 6.6	17.4 ± 2.4
	Creatinine	67 ± 11.4	69.3 ± 11.4	66.1 ± 9.4
Group-IV	SGPT	16.4 ± 10.3	13.8 ± 6.2	12.7 ± 4.7
	Creatinine	64.5 ± 1.4	65 ± 7.7	55.8 ± 4.4
Group-V	SGPT	20.9 ± 6.5	19.9 ± 6.4	24.2 ± 10.3
	Creatinine	61.4 ± 9.4	62.7 ± 9.2	66.1 ± 13.1

Note. Group-I: 250 mg PB b.d. + 5000 IU vitamin D_3 o.d.; Group-II: 500 mg PB b.d. + 5000 IU vitamin D_3 o.d.; Group-III: 1000 mg PB b.d. + 5000 IU vitamin D_3 o.d.; Group-IV: 500 mg PB b.d.; Group-V: 5000 IU vitamin D_3 o.d. Data expressed as Mean ± Standard Deviation. One Way Analysis of Variance (ANOVA) method was used. PB- Phenylbutyrate; SGPT- *S*erum Glutamate Pyruvate Transaminase; b.d. - twice daily; o.d. - once daily. SGPT values are given as IU/L; creatinine values are given as mg/L. The normal range for SGPT and creatinine are (0.01-56 IU/L) and (45-106 µmol/L) respectively. One Way Analysis of Variance (ANOVA) method was used.
